# Evaluation of Pleural Vents Inserted by Radiologists Versus Intercostal Chest Drains Managed by Respiratory Physicians for Post-CT-Guided Lung Biopsy Pneumothorax at Glan Clwyd Hospital, Wales

**DOI:** 10.7759/cureus.97248

**Published:** 2025-11-19

**Authors:** Sam Doherty, Elen Jones, Rajiyah Hussain, Diab Alsouki, Lawi Suissa, Ahmed Abou-Haggar

**Affiliations:** 1 Medicine, Betsi Cadwaladr University Health Board, Bodelwyddan, GBR

**Keywords:** ct guided biopsy, intercostal chest tube drain, pleural vent, pneumothorax management, radiologist-performed biopsy

## Abstract

Introduction

CT-guided biopsy (CTGB) of the lung is a commonly performed, minimally invasive procedure used to obtain tissue samples from suspected malignant lung lesions. Pneumothorax is a recognised complication of CTGB. Management typically involves observation, intercostal chest drain insertion, or pleural vent placement. Traditional intercostal chest drains often require unplanned hospital admission, whereas pleural vents allow patient mobility and may reduce inpatient care.

Objective

The primary aim of this study was to compare hospital length of stay in patients managed with pleural vents inserted by radiologists versus intercostal chest drains managed by respiratory physicians for post-CTGB pneumothorax.

Materials and methods

A retrospective analysis was conducted of 98 scheduled CT-guided biopsies performed over a one-year period at Glan Clwyd Hospital, Wales. Seventeen cases were excluded due to lesion resolution, procedure abandonment, biopsies outside the lungs, medically unfit for biopsy, or inaccessible biopsy sites, leaving 81 biopsies for analysis.

Results

Among the 81 patients, 39 experienced complications, including 30 pneumothoraces and nine haemothoraces. Nine patients with pneumothorax required intervention: four were managed with pleural vents inserted by the operating radiologist, and five received intercostal chest drains inserted by the respiratory team. The modal length of stay was one day for pleural vent patients and three days for chest drain patients.

Conclusion

Pleural vents were associated with a shorter modal length of hospital stay (one day) compared with intercostal chest drains (three days) for post-CTGB pneumothorax. Whilst limited by its single-centred, observational nature, this study provides useful real-world insight into current pneumothorax management by radiologists and respiratory physicians. Further prospective multicentre research is needed to validate the potential advantage of pleural vents over intercostal chest drains and support the development of standardised protocols and potential outpatient pathways for CTGB pneumothorax management.

## Introduction

Lung cancer is the third most common cancer in the UK and remains the leading cause of cancer-related mortality, accounting for approximately 35,000 deaths annually [1]. Consequently, early identification through biopsy is vital for improving long-term survival. Biopsy provides tissue samples from suspected malignant lung lesions for histological evaluation. Several approaches to biopsy exist, including CT-guided percutaneous transthoracic needle biopsy, electromagnetic navigation bronchoscopy (ENB), and endobronchial ultrasound-guided transbronchial needle aspiration (EBUS-TBNA) [2]. At Glan Clwyd Hospital, Wales, radiologists and respiratory physicians jointly assess patient suitability for CT-guided biopsy (CTGB) during multidisciplinary team meetings. CTGB is a minimally invasive technique with a particularly high degree of diagnostic accuracy, reported to be up to 97% [3]. CTGB is especially useful for lesions located peripherally, which would otherwise be difficult to sample using alternative methods [4].

Pneumothorax is a recognised complication associated with CT-guided biopsy, with an incidence rate of approximately 26% [5]. A pneumothorax occurs when air enters the pleural space - the area between the lung and chest wall - resulting in partial or total lung collapse [6]. At Glan Clwyd Hospital, Wales, pneumothoraces are currently managed in three main ways: (i) observation, (ii) insertion of an intercostal drain attached to an underwater seal, or (iii) placement of a pleural vent. The choice of management depends on various factors, including the size of the pneumothorax, the timing of its occurrence, and the radiologist performing the biopsy.

Management with a conventional intercostal chest drain often necessitates unplanned hospital admission for monitoring and intervention. This is largely due to potential complications associated with outpatient chest drain management, including pneumonia, empyema, and high readmission rates [7].

The use of pleural vents has recently been introduced at Glan Clwyd Hospital, Wales, as a means of managing iatrogenic pneumothorax following biopsy. The Rocket® Pleural Vent™ is an 8F ambulatory chest drain designed for the treatment of pneumothorax. It enables patients to remain fully mobile and may reduce the need for inpatient management. Extended hospital stays have been associated with an increased risk of hospital-acquired complications, including infections, venous thrombosis, depression, physical deconditioning, and even mortality [8]. These complications can significantly impact both patient outcomes and healthcare costs [8].

There is currently limited evidence evaluating the use of pleural vents for iatrogenic post CTGB pneumothorax. This study primarily aims to compare the length of stay (LoS) of CTGB-related pneumothorax patients managed with pleural vents inserted by radiologists, compared with traditional intercostal chest drains managed by respiratory physicians.

This article was previously presented as a conference abstract titled “Evaluation of Pleural Vents vs Indwelling Chest Drains for Post CT-Guided Biopsy Pneumothorax” at the Medicine 2025: The Future of Medicine, Royal College of Physicians (RCP) Conference on June 2 2025, and subsequently published as a conference abstract only in Clinical Medicine in July 2025 (ScienceDirect supplement; doi.org/10.1016/j.clinme.2025.100397). This manuscript includes the full data, extended methods, results, discussion, and conclusions that were not previously published.

## Materials and methods

Study Design and Setting

A retrospective analysis was conducted of all CTGB scheduled at Glan Clwyd Hospital in North Wales, over a one-year period between 2 November 2023 and 19 November 2024, following the introduction of pleural vents. The aim of this analysis was to primarily evaluate the length of stay between pneumothorax managed with pleural vents and intercostal chest drains within a real-world clinical setting.

Patient Identification and Data Collection

All CTGB procedures performed during the study period were identified through interrogation of departmental procedure logs. Case details were cross-referenced with procedure reports on Synapse and the Picture Archiving and Communication System (PACS) to ensure data completeness and accuracy.

For each identified case, relevant clinical and procedural data were extracted as per Table 1. All data were anonymised prior to analysis in accordance with institutional data protection policies and the General Data Protection Regulation (GDPR).

**Table 1:** Data collected

Demographic, clinical and procedural data collected
Patient demographics (age, sex)
Lesion characteristics (size, location, emphysema, crossing interlobar fissure)
Procedural details (Operator experience level, Biopsy needle distance to reach site)
Post-procedural complications (pneumothorax or haemorrhage)
Intervention required for complications (pleural vent or intercostal chest drain, anti-fibrinolytic therapy, blood transfusion)
Length of Stay (LoS) in hospital

Study Population and Inclusion and Exclusion Criteria

A total of 98 CTGB procedures were scheduled during the study period. After application of the predefined inclusion and exclusion criteria (Tables 2-3), 81 CTGB procedures were available for final analysis. This cohort constituted the study population for subsequent evaluation of the impact of pleural vent use and intercostal chest drains on LoS.

**Table 2:** Inclusion criteria

Inclusion criteria	Number of patients (n)	Percentage (%)
CT-guided lung biopsy performed between 2 November 2023 and 19 November 2024	98	100%
Target lesions located within the lung parenchyma	98	100%
Adequate clinical imaging and data available	98	100%
Full procedural and post procedural documentation available	98	100%

**Table 3:** Exclusion criteria

Exclusion criteria	Number of patients (n)	Percentage (%)
Resolution of the lesion prior to the procedure	4	4%
Abandonment of procedure or non-attendance	8	8%
Biopsy performed on anatomical structures outside the lungs	3	3%
Patient deemed unfit for procedure	1	1%
Inaccessible biopsy site	1	1%

Outcome Measures and Data Analysis

The primary outcome measure was LoS in post-procedural pneumothorax patients managed with pleural vent or intercostal chest drain. Descriptive statistics were used to summarise patient demographics, lesion characteristics, and procedural outcomes. Continuous variables were reported as counts and percentages.

Ethical Considerations

The study was registered with the Glan Clwyd Hospital Audit and Clinical Governance Department and conducted as part of a service evaluation project. As a retrospective analysis of anonymised data, formal ethical committee approval and individual patient consent were not required under the NHS Research Authority guidelines.

## Results

A total of 81 patients who underwent CTGB were included in the analysis. The cohort comprised 40 males (49%) and 41 females (51%), with a mean age of 70 years (range: 44-89 years). The balanced gender distribution allowed for accurate analysis; uneven distribution may have influenced anatomical sites available for biopsy.

Of the 81 patients, 39 (48%) experienced a complication. Pneumothorax was the most commonly occurring complication in 30 patients (37%), followed by haemorrhage, which occurred in nine patients (11%), as shown in Table 4.

**Table 4:** Frequency and percentages of complications

Complication	Number of patients (n)	Percentage (%)
Total complication amount	39	48
Pneumothorax	30	37
Haemorrhage	9	11

Among the 30 patients with pneumothorax, the majority were managed with routine observation due to the size and asymptomatic nature of pneumothorax, as per national guidelines, as shown in Table 5. A total of nine patients (30%) who experienced pneumothorax required active intervention in the form of either pleural vent or intercostal chest drain. Four patients (13%) were managed with pleural vents inserted at the time of biopsy by the radiologist, and five patients (17%) required an intercostal chest drain placed by the respiratory team after biopsy on inpatient wards seen in Table 5. Of the nine haemorrhage cases, two patients were treated with tranexamic acid (22%); none of these patients were receiving anticoagulant therapy, and no blood transfusions were required.

**Table 5:** Management of pneumothorax

Type of management	Number of patients (n)	Percentage (%)
Observation	21	70
Pleural vent	4	13
Intercostal chest drain	5	17

After a CTGB, patients underwent a chest X-ray to assess for iatrogenic pneumothorax. The chest X-rays related to the nine pneumothorax patients who required intervention were reviewed by a respiratory physician. The following criteria were used to determine suitability for pleural vent management. The use of a pleural vent was deemed unsuitable for patients who were clinically unstable, had pneumothorax greater than 2 cm, had tension pneumothorax, had significant pleural fluid (e.g., effusion or empyema), had subcutaneous emphysema, or had difficult chest wall anatomy. Based on these criteria, the physician concluded that all nine cases reviewed could have been initially managed with a pleural vent and monitoring.

Factors associated with an increased risk of pneumothorax following CTGB were recorded for their potential impact on patient outcomes. The average tumour size was 24.9 mm, the mean needle distance to the site of biopsy of 62.3 mm, and emphysema presence (n=29 patients, 36% of the total cohort), and the needle path crossed interlobar fissures in three cases.

**Figure 1:**
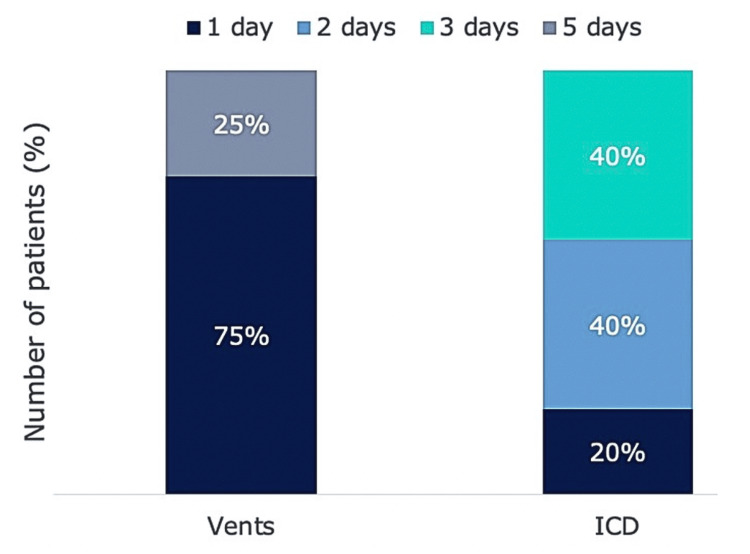
Length of stay in days of post CTGB pneumothorax patients managed with pleural vents vs intercostal chest drains CTGB (CT guided biopsy), ICD (Intercostal chest drains), Vents (Rocket® Pleural Vent™)

Measuring the LoS, in days, was the primary outcome of this study, comparing pleural vents with intercostal chest drains. Patients discharged on the same day were assigned a LoS of one day, and overnight admissions were considered to be a LoS of two days. The pleural vent group demonstrated a shorter modal LoS (one day) compared with the intercostal chest drain group (three days), as shown in Figure 1. Of the pleural vents group, only one patient had a LoS over one day. This patient had medical conditions unrelated to the use of pleural vents, such as newly diagnosed atrial fibrillation, which contributed to a prolonged hospital stay. This is shown in Figure 1 as 25% of the vents group staying for five days. 

**Figure 2:**
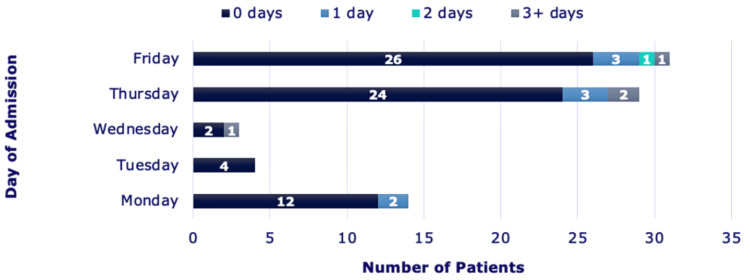
Day of CT-guided biopsies vs number of patients with associated LoS LoS (Length of Stay). Total: 81 biopsies

Figure 2 shows the day of the week on which each biopsy was performed and the subsequent LoS for each patient. As illustrated, the majority of CT-guided biopsies occurred later in the workweek, with 29 patients (36% of total biopsies) on Thursday and 31 patients (38% of total biopsies) on Friday, and admissions followed a similar trend.

## Discussion

In our study, the modal LoS for pleural vent patients was one day, compared with three days for intercostal chest drains. Our observational findings are consistent with previous literature, suggesting that pleural vents may reduce inpatient burden in the management of pneumothorax [9-14].

Pleural vents are designed for ambulatory management, allowing patients to remain mobile and to be safely discharged once imaging confirms adequate lung re-expansion and the absence of an air leak [9]. Unlike traditional intercostal chest drains, they do not require continuous underwater seal monitoring or inpatient observation [9].

Reduced inpatient stay minimises exposure to hospital-acquired complications, improves patient satisfaction, and lowers overall healthcare costs [9-14]. The use of pleural vents as a practical tool for outpatient management of pneumothorax, in clearly defined patient groups, is supported by previous randomised controlled trials and single-centre studies [13,14]. These advantages may be of particular relevance for high-volume centres performing frequent CT-guided biopsies, where even small reductions in admission duration can translate into significant resource savings and improved bed availability.

In our study, all pleural vents were inserted by the operating radiologist, whereas all intercostal chest drains were placed by respiratory physicians. This distinction in procedural practice reflects differing levels of familiarity and confidence with pleural vent devices among healthcare teams. This highlights a potential need for further training and interdisciplinary collaboration to enhance awareness, competency, and confidence in the use of pleural vents as an alternative to traditional intercostal chest drains.

The overall pneumothorax rate in our cohort was 37%, which is higher than the average incidence reported in the literature (approximately 26%) [5]. This increased rate may, in part, be attributed to several non-modifiable risk factors, such as patient age, presence of emphysema, lesion size, depth, and needle trajectories crossing interlobar fissures. Emphysema, present in 36% of our cohort, is a well-established risk factor known to substantially increase the likelihood of pneumothorax following CT-guided lung biopsy [5,15]. Emphysematous lungs are structurally fragile, characterised by disrupted alveolar architecture and reduced elastic recoil, which makes them particularly prone to air leakage when punctured [15]. The higher incidence observed in our study may also reflect institutional or procedural variations, including differences in operator technique, needle trajectory, number of pleural passes, biopsy system used, or patient positioning.

It was also observed that most CTGBs were performed later in the week, 36% on Thursday and 38% on Friday, leading to a clustering of admissions. This scheduling pattern may have inadvertently contributed to prolonged hospital stays. Patients biopsied late in the week are perhaps more likely to remain in hospital over the weekend due to reduced availability of medical staff to review and discharge patients with intercostal chest drains specifically. To optimise patient flow and resource utilisation, it may be beneficial to schedule CT-guided lung biopsies earlier in the week, when full multidisciplinary support is available. Such an approach could facilitate timelier discharge, reduce bed occupancy, and minimise the impact of non-clinical factors on LoS.

Our study has several limitations that should be acknowledged. First, its retrospective, single-centre design introduces potential selection and reporting bias, which may influence the observed outcomes. Variability in operator experience, procedural technique, and decision-making regarding pleural vent versus intercostal drain use was not standardised and may have affected results. Additionally, as previously mentioned, the LoS may have been affected by non-clinical influences such as weekend staffing levels, discharge policies, and departmental workflow patterns.

Finally, the small number of patients who required active intervention for pneumothorax resulted in very small comparison groups for pleural vents (n=4) and intercostal chest drains (n=5). This restricted sample size limits the robustness and generalisability of the findings. In addition, no formal statistical analysis was performed to compare outcomes between the two groups, meaning that observed differences in LoS cannot be interpreted as statistically significant and may be influenced by chance or confounding factors.

Despite these constraints, the study offers valuable real-world observational insight into current practice in the management of CTGB-related pneumothorax. The observational nature of the work captures how pleural vents and intercostal drains are used in routine clinical settings, reflecting genuine patient pathways, operational pressures, and clinician decision-making that may not be fully represented in controlled research environments.

Additionally, the study highlights patterns of practice that could be optimised, such as biopsy scheduling and interdisciplinary collaboration, thereby informing quality improvement initiatives and guiding future prospective research aimed at standardising and refining pneumothorax management strategies. This study, therefore, offers useful baseline data for hospitals considering service improvement.

## Conclusions

Pleural vents may potentially offer a resource-efficient alternative to traditional intercostal chest drains for managing pneumothorax following CTGB in selected patient groups. Their use may be associated with shorter hospital stays, offering clinical and economic advantages. However, the retrospective, single-centre design and small sample size limit the strength of these conclusions.

Despite this, our results provide meaningful real-world insight into the management of CTGB-related pneumothorax by radiologists and respiratory physicians. Future prospective multicentre studies are needed to confirm these potentially highlighted benefits, refine patient selection, and establish standardised procedural protocols to further enhance safety, efficiency, and the feasibility of outpatient care in the management of CTGB-related pneumothorax.
